# Design and screening of novel 1,2,4-Triazole-3-thione derivatives as DCN1 inhibitors for anticardiac fibrosis based on 3D-QSAR modeling and molecular dynamics

**DOI:** 10.3389/fphar.2025.1590711

**Published:** 2025-06-27

**Authors:** Wengong Bian, Yaxin Guo

**Affiliations:** Department of Anesthesiology, Shandong Provincial Third Hospital, Jinan, Shandong, China

**Keywords:** DCN1, 3D-QSAR modeling, molecular docking, molecular dynamics simulation, anticardiac fibrotic

## Abstract

**Objective:**

Defective in cullin neddylation 1 (DCN1) plays a pivotal role in anticardiac fibrosis by interacting with UBC12 and catalyzing cullin neddylation, which activates cullin-RING E3 ligases (CRLs). As a key modulator of anticardiac fibrosis, DCN1 has emerged as an attractive target for therapeutic intervention. The aim of this study is to design and evaluate novel DCN1 inhibitors using a combination of three-dimensional quantitative structure-activity relationship (3D-QSAR) modeling, molecular docking, and molecular dynamics simulations.

**Methods:**

A dataset of 47 derivatives was employed to construct Comparative Molecular Field Analysis (COMSIA) model, incorporating steric, electrostatic, hydrophobic, hydrogen bond donor, and acceptor fields to accurately predict compound activity. In silico molecular docking studies, selected compounds were docked with the target protein to evaluate their binding affinity. Additionally, molecular dynamics simulations were performed to assess the stability of the compounds, followed by energy decomposition analysis was used to identify key residues contributing to binding.

**Results:**

The comparative molecular similarity index analysis (COMSIA) model achieved a cross-validated q^2^ of 0.553, a non-cross-validated r^2^ of 0.959, and an $R_{ext}^{2}$ value of 0.766, demonstrating good accuracy and stability in predicting the activity of the compounds. The top compound exhibited a predicted pIC50 of 9.674 and showed strong binding affinity in molecular docking. Molecular dynamics simulations confirmed the stability of the compound at the binding site, while energy decomposition analysis identified key residues essential for binding interaction.

**Conclusion:**

This study successfully designed and evaluated novel DCN1 inhibitors using an integrated approach that combines 3D-QSAR modeling, molecular docking, and molecular dynamics simulations. The findings provide an effective computational platform for the design of DCN1 inhibitors and lay a solid foundation for the development of drugs targeting anticardiac fibrosis.

## 1 Introduction

Cardiovascular diseases (CVDs) remain the leading cause of morbidity and mortality worldwide, accounting for approximately 17.9 million deaths annually. According to the World Health Organization (WHO), this represents 32% of all global deaths, with ischemic heart disease and stroke being the predominant contributors (Frangogiannis, 2021). The burden of CVDs is particularly high in low and middle income countries. Cardiac fibrosis plays a critical role in the progression of heart failure, hypertensive heart disease, and cardiomyopathies (Frangogiannis, 2017). About 40%–50% of heart failure patients can be into myocardial fibrosis. Fibrosis contributes to diastolic dysfunction, arrhythmias, and impaired contractility, making it a key therapeutic target in cardiology (Segura et al., 2014).

Protein neddylation is one type of posttranslational modifications that regulates the activity of the substrate proteins. Neddylation modification is catalyzed by NEDD8-activating enzyme (NAE, E1), NEDD8-conjugating enzyme (E2), and NEDD8 ligase (E3) to attach NEDD8, an ubiquitin-like molecule, to a lysine residue of a substrate protein (Lu and Yang, 2020). Targeting protein-protein interactions of the neddylation complexes has been pursued as a potential strategy to selectively inhibit the activity of individual cullin-RING ligases (CRLs) (Huang et al., 2023). Analysis of the co-crystal structures of DCN1, a co-E3 for neddylation, and its binding partners UBC12 (a neddylation E2) suggested that it may be amenable for the design of potent, small-molecule inhibitors. DCN1 and DCN2 share homology but differ in their interaction networks. DCN1 is critical for cullin1/3 neddylation, while DCN2 is implicated in cullin4/5 neddylation. The hydrophobic pocket in DCN1 that accommodates the N-acetyl-Met residue of UBC12 (a key E2 enzyme) has structural variations compared to DCN2 (Zheng et al., 2021). DCN1, a co-E3 ligase, interacts with UBC12 and activates cullin-RING ligases (CRLs) by catalyzing cullin neddylation (Paccez et al., 2024). Although DCN1 has been recognized as an important therapeutic target for human diseases, its role in the cardiovascular area remains unknown. DCN1 can upregulate in isolated cardiac fibroblasts (CFs) treated by angiotensin (Ang) II and in mouse hearts after pressure overload (Chatzifrangkeskou et al., 2016). Then, structure-based optimizations for DCN1-UBC12 inhibitors of DN-2 were performed. DN-2 specifically targeted DCN1 at molecular and cellular levels as shown by molecular modeling studies, HTRF, cellular thermal shift and co-immunoprecipitation assays (Fang et al., 2019). Importantly, DN-2 effectively reversed Ang II-induced cardiac fibroblast activation, which was associated with the inhibition of cullin 3 neddylation. Studies indicate a potentially role of DCN1 inhibition for anticardiac fibrotic effects. There is still lack of potent DCN1 inhibitor with high selectivity and good pharmacokinetic property (He et al., 2023). Therefore, novel and efficient DCN1 inhibitors with drug-like properties remain to be urgently needed to explore more potential biological functions, especially anticardiac fibrotic effect *in vivo* (He Z. et al., 2024).

Three-dimensional quantitative structure-activity relationship (3D-QSAR) is a powerful computational approach used in drug design and screening, offering improved predictive capabilities, a deeper understanding of molecular interactions, and the potential to accelerate the drug discovery process. 3D-QSAR takes into account the three-dimensional arrangement of atoms and molecular interactions. By considering the three-dimensional arrangement of atoms and molecular interactions, This allows for more precise predictions about how minor structural modifications to a compound can impact its biological activity. This work will apply the 3D-QSAR and molecular dynamics simulation to design new structures of 1,2,4-Triazole-3-thione derivatives of DCN1 inhibitors (He Z-X et al., 2024).

## 2 Materials and methods

### 2.1 Data sets and biological activity

In this study, a dataset of 47 derivatives from the literature was utilized for 3D-QSAR model building. The IC50 values of these compounds were converted into pIC50 values [pIC50 = −log (IC50) +9] and served as the dependent variable for further analysis. Table 1 shows the structures of all compounds, along with their IC50 and pIC50 values. The dataset was randomly split into a training set consisting of 38 compounds which were applied for building 3D-QSAR model. The test set involves 9 compounds that were used as independent samples for model validation. The ratio of compounds in the training set to the test set was approximately 4:1 (Li et al., 2016).

**TABLE 1:** 47 structures and the experiment IC50.

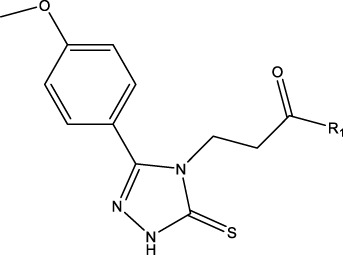 9–12			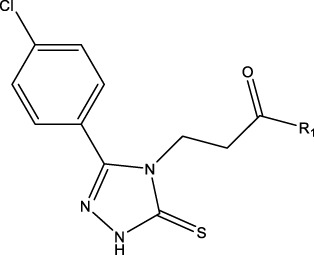 17–20		
Compd	R1	IC50 (nM)^a^	pIC50	COMFA^a^ Pred	COMSIA^b^ Pred
9	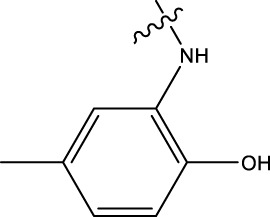	1,460.2	5.7851	5.747	5.839
10	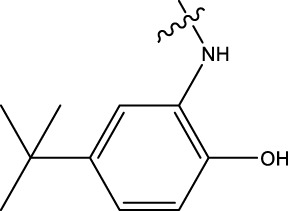	2,321.13	5.6343	5.715	5.737
11^c^	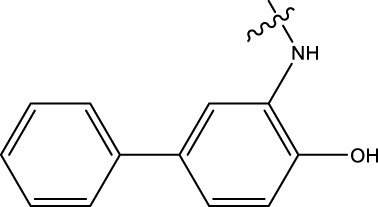	1,110.14	5.9546	5.547	5.619
12	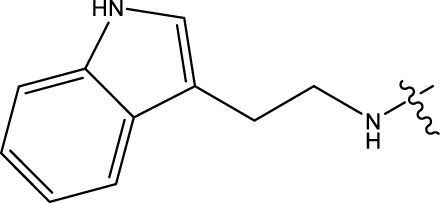	2,280.24	5.6416	5.647	5.816
17	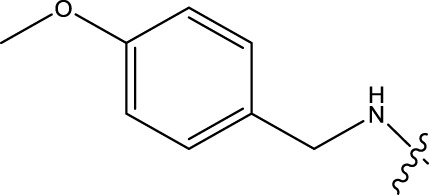	650.16	6.187	6.13	6.018
18	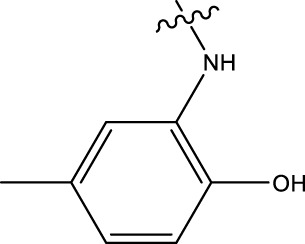	195.04	6.7099	6.739	6.742
19	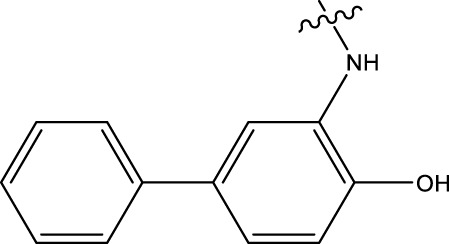	329.96	6.4815	6.403	6.438
20^c^	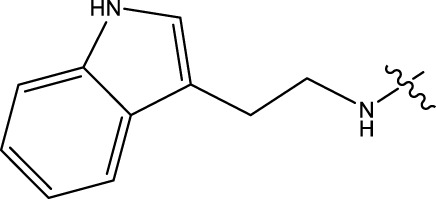	2,170.21	5.6635	5.536	5.487

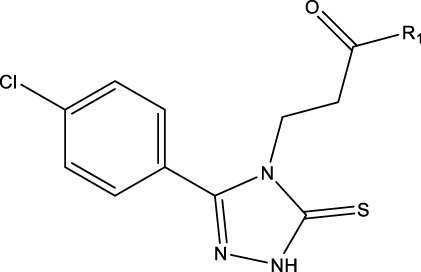					
Compd	R1	IC50(nM)^a^	pIC50	COMFA Pred	COMSIA Pred
25	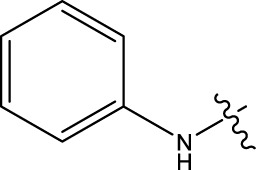	545.15	6.2635	6.16	7.09
26	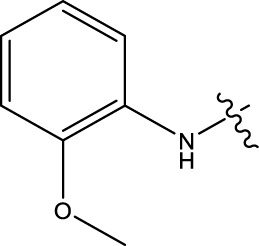	883.62	6.0537	6.055	5.758
27	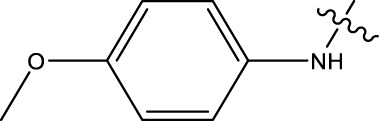	90.87	7.0416	7.093	6.959
28	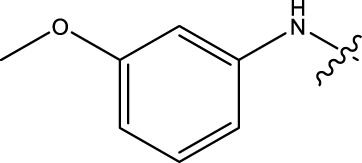	77.62	7.11	7.108	7.068
30	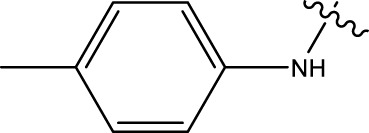	50.51	7.2966	7.346	7.09
31	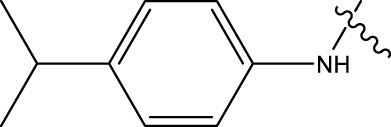	12.82	7.8921	7.869	7.698
32	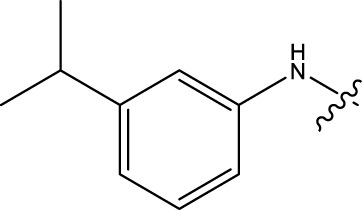	15.65	7.8055	7.717	8.116
33	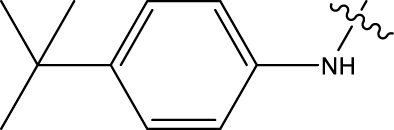	8.77	8.057	9.18	8.783
34^c^	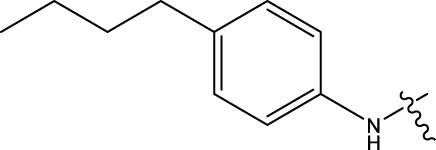	12.31	7.9097	7.535	7.161
35	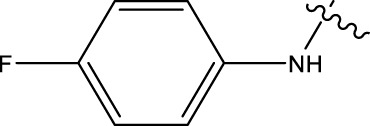	130.22	6.8853	6.942	6.949
36	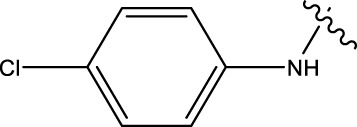	69.40	7.1586	7.136	7.098
37^c^	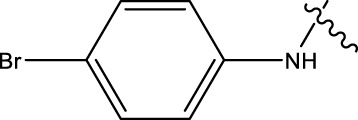	25.65	7.5909	7.859	7.284
38	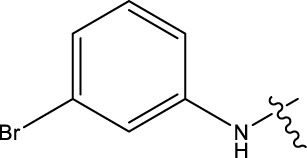	22.17	7.6542	7.711	7.615
39	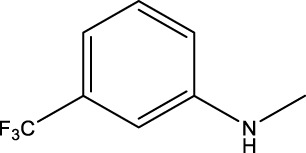	2.96	8.5287	8.317	8.208
40^c^	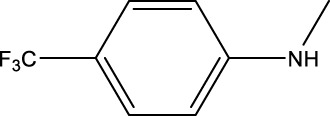	10.57	7.9759	7.249	7.166
41	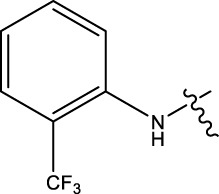	100.11	6.9995	6.974	7.002
42	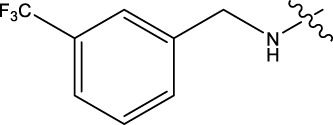	43.06	7.3659	7.686	7.819
43	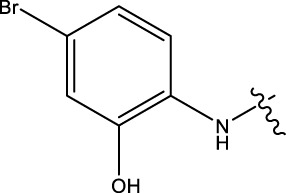	208.86	6.6801	6.633	6.591
44	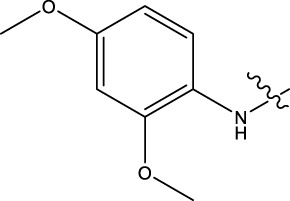	840.33	6.0756	6.034	6.039
45	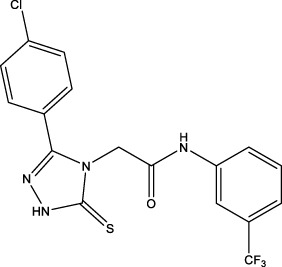	27.42	7.5619	7.578	7.5
46	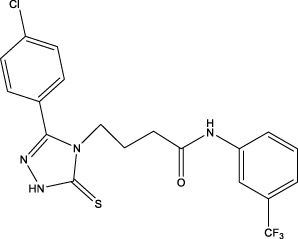	45.16	7.3452	7.307	7.237

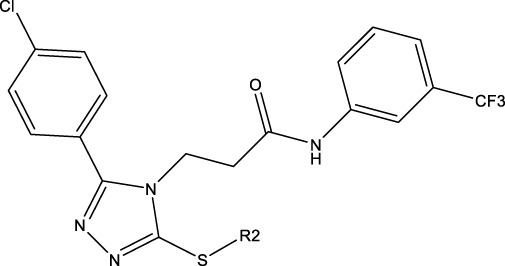					
Compd	R1	IC50(nM)^a^	pIC50	COMFA Pred	COMSIA Pred
47^c^	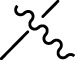	1,630.11	5.7878	5.556	5.014
48	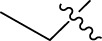	2,203	5.657	5.597	5.675
49	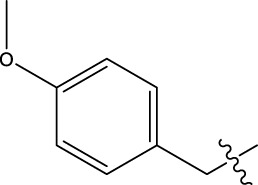	4,114	5.3857	5.352	5.383
50	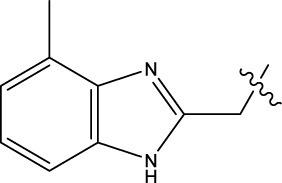	7,114	5.1479	5.233	5.034
53	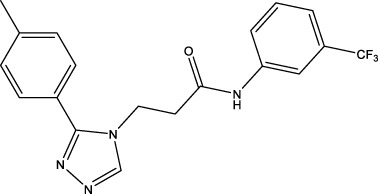	15850	4.8	4.912	4.826
54^c^	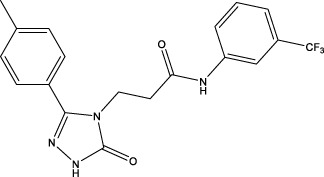	7,580	5.1203	5.437	5.142

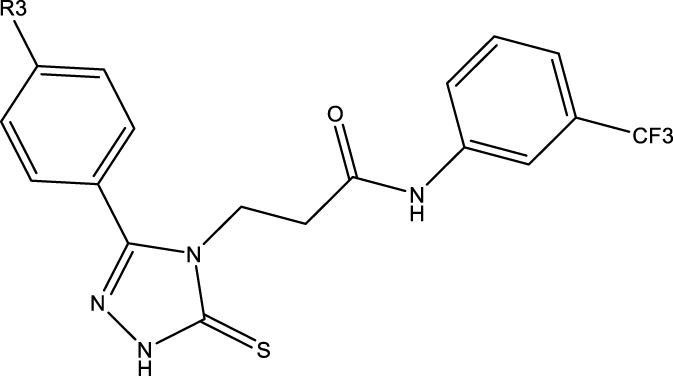					
Compd	R1	IC50 (nM)^a^	pIC50	COMFA Pred	COMSIA Pred
55	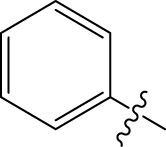	15.27	7.8162	7.707	7.687
56	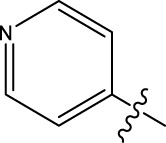	221.19	6.6552	6.77	6.728
57	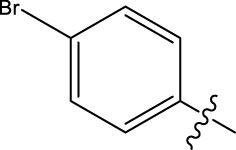	32	7.6043	7.649	7.726
58^c^	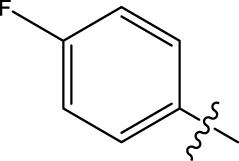	18.91	7.7233	7.934	7.058
59	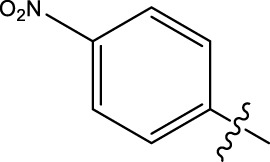	46.17	7.3356	7.175	7.324
60	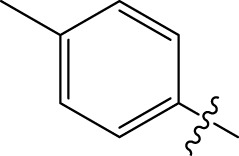	9.42	8.0259	8.066	8.102
61	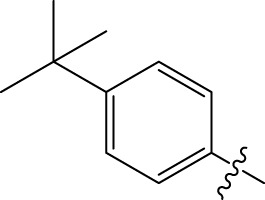	7.85	8.1051	8.054	8.042
62	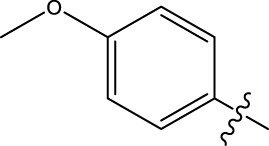	14.06	7.852	7.861	7.868
63^c^	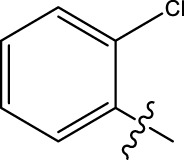	28.84	7.54	7.378	7.373
64	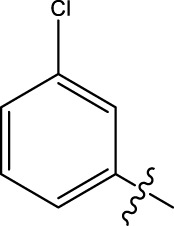	19.92	7.7009	7.659	7.705
65	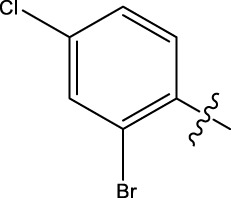	5.41	8.2668	8.406	8.227
66	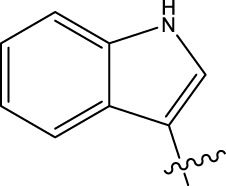	9.39	8.0273	7.983	8.113

^a^ Comparative molecular field analysis (COMFA).

^b^ Comparatie molecular similarity index analysis (COMSIA).

^c^ Test set.

### 2.2 Molecular modeling

All computational analyses in this investigation were executed utilizing the Sybyl-X 2.1 platform. QSAR framework embedded in Sybyl was applied to delineate associations between structural derivatives and their inhibitory potency (IC50). Initial compound architectures were generated via ChemDraw Professional, followed by geometric refinement within Sybyl’s molecular workspace. Structural energy optimization was achieved through the Tripos force field coupled with the Powell conjugate gradient method, employing a termination criterion of 0.05 kcal/(mol·Å) and a cap of 10,000 optimization cycles (Buch et al., 2010). This protocol guaranteed the selection of the lowest-energy molecular configuration, which served as the foundational geometry for subsequent comparative molecular similarity index analysis (COMSIA) (Putz et al., 2016). To refine electrostatic modeling, Gasteiger-Hückel partial charge assignments were systematically applied across all atomic sites.

### 2.3 Conformational sampling and alignment

**FIGURE 1:**
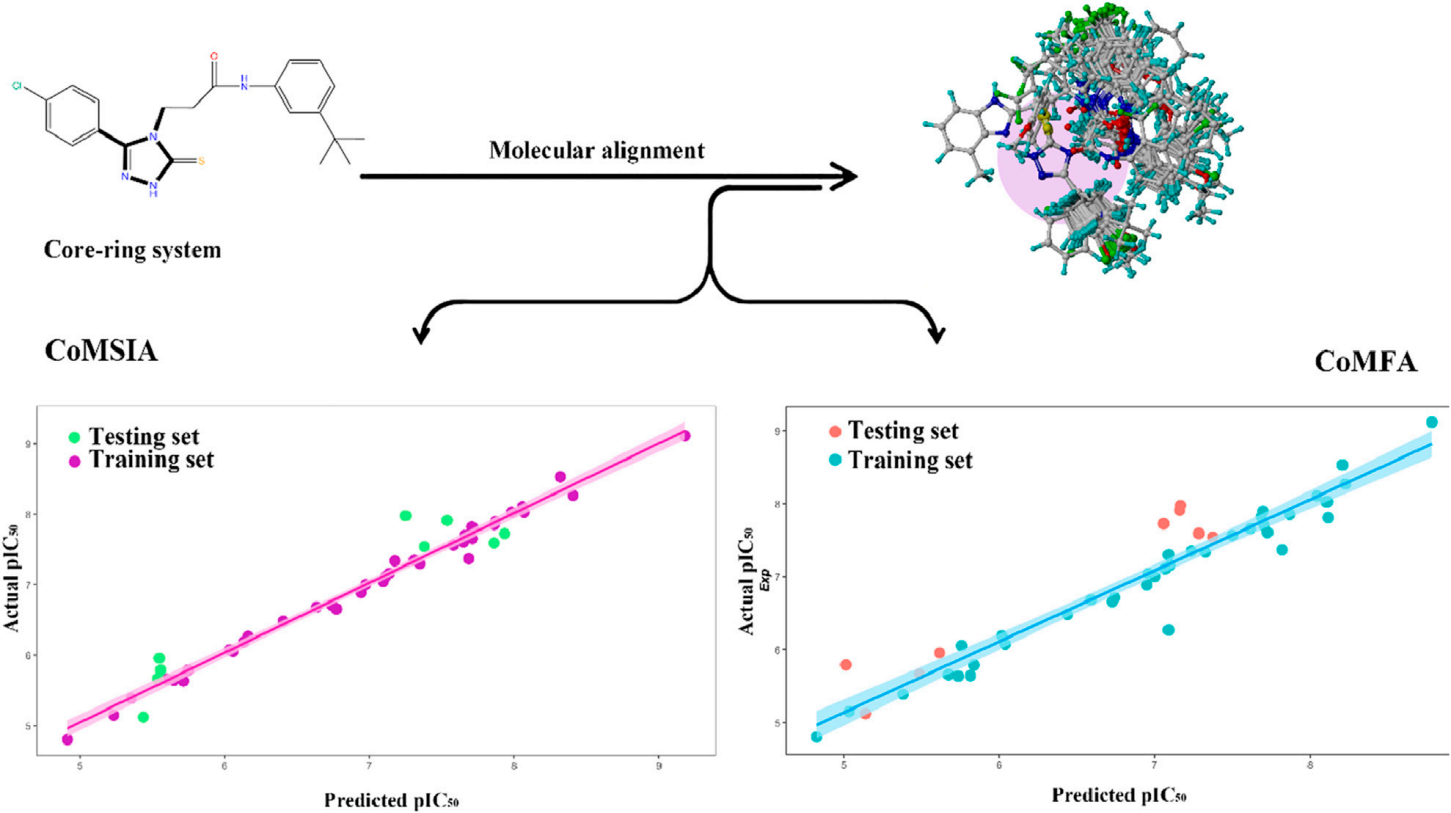
Regression analysis comparing the model-predicted pIC50 values with the actual pIC50 values, COMFA values, and COMSIA values.

The predictive reliability of COMSIA is inherently dependent on molecular alignment methodologies. In this investigation, ligand structural alignment was performed using the COMSIA module, with the analytical cohort comprising compounds featuring a conserved structural motif (Figure 1). A maximum common substructure (MCS) driven alignment protocol integrated into COMSIA was adopted for spatial superposition. Though compound 33 has the higher inhibitor (Figure 1), it has lower toxicology. Therefore, this compound was selected as the reference template for systematic alignment. Subsequent molecular superimposition of all derivatives was executed within the Sybyl computational platform, ensuring consistency in spatial orientation for comparative analysis (Akamatsu, 2002; Lanka et al., 2023).

### 2.4 COMSIA model study

The COMSIA simulations were conducted within the Sybyl-X 2.1 suite under predefined settings, incorporating Lennard-Jones potentials and Coulombic field gradients. Five physicochemical descriptors steric (S), electrostatic (E), hydrophobic (H), hydrogen bond donor (D), and hydrogen bond acceptor (A) were systematically evaluated to characterize ligand-receptor binding mechanisms. The attenuation coefficient (α) remained fixed at 0.3 to maintain consistency with prior protocols.

3D-QSAR model was constructed by partial least squares (PLS) regression (Cramer et al., 1988), correlating structural features with experimental pIC50 values. Model validation employed leave-one-out (LOO) cross-validation, interactively excluding individual compounds to predict their activities using the remaining dataset. This approach optimized the number of latent components (NLC) and derived the cross-validated correlation coefficient (q^2^) by minimizing the prediction error sum of squares. Subsequently, a non cross validated analysis using the finalized NLC yielded the coefficient of determination (r^2^), standard error of estimation (SEE), and F-test statistic, ensuring statistical robustness of the model.

### 2.5 Molecular docking

Computational ligand-receptor interaction analysis was executed via the Sybyl-X 2.1 suite to map binding modalities and identify bioactive conformers. Structural coordinates of DCN1-specific inhibitory complexes were sourced from the RCSB PDB repository for molecular docking simulations (PDB ID: 5UFI) (Morris and Lim-Wilby, 2008). The crystallographic structure underwent preprocessing by eliminating non-protein components (including solvent molecules) and retaining only the macromolecular framework. Protonation states were optimized by introducing hydrogen atoms at polar sites, followed by charge parameterization using the Kollman united atom force field to prepare the system for energy calculations (Fan et al., 2019).

The active site was delineated using the Sybyl-X 2.1 computational suite, with ProtoMol parameters configured to default settings (expansion coefficient: 0; distance cutoff: 0.5 Å) for spatial characterization. For each ligand, the top-scoring 20 poses were retained and prioritized according to docking energy metrics. Optimal ligand-receptor configurations were subjected to molecular interaction profiling to map critical binding features, supported by visual representations of key hydrogen bonding networks, electrostatic interfaces, and hydrophobic contacts.

### 2.6 Molecular dynamics simulation

Atomistic conformational dynamics of prioritized protein-ligand complexes were explored via GROMACS 2023.2 with the CHARMM36m force field (Huang et al., 2017), enabling nanoscale resolution of structural transitions. Ligand topology was parameterized using the CGenFF server (Vanommeslaeghe and MacKerell, 2012), and charge neutrality was achieved by incorporating monovalent counterions (Na^+^/Cl^−^). Solvation employed the CHARMM-optimized TIP3P water model, with terminal residues manually assigned as NH_3_^+^ (N-terminus) and COO^−^ (C-terminus) to override default settings incompatible with methionine-initiated polypeptides.

The system underwent potential energy optimization through sequential steepest descent and conjugate gradient algorithms. Non-bonded interactions were partitioned into short range (<10Å) and long range components, the latter resolved via the Particle Mesh Ewald (PME) method, while LINC constraints managed covalent bonds. Thermostatic control (300 K) utilized the V-rescale algorithm, and isotropic pressure coupling (1 bar) was maintained via the Parrinello-Rahman barostat. Following energy relaxation, sequential NVT (100 ps) and NPT (100 ps) equilibration phases ensured thermodynamic stability, preceding a 100-ns production simulation with trajectory snapshots recorded at 2-fs intervals.

Post-simulation analytic included conformational stability (RMSD), residue flexibility (RMSF), and intermolecular H-bond dynamics, processed through native GROMACS utilities. Ligand-binding energetics were quantified via the molecular mechanics-poisson-boltzmann surface area (MM-PBSA) framework, decomposing free energy into van der Waals, electrostatic, solvation, and entropic terms to guide structure-activity optimization. Computational binding affinities were benchmarked against experimental data to validate predictive reliability, with gmx_MMPBSA extracting energy metrics from the terminal 100-ns trajectory window for 3C protease-ligand systems (Valdés-Tresanco et al., 2021).

## 3 Results and discussion

### 3.1 COMSIA statistical results

The statistical analysis of the optimal COMSIA model identified five crucial molecular fields: steric, electrostatic, hydrophobic, hydrogen bond donor, and hydrogen bond acceptor (Klebe and Abraham, 1999). A total of 16t models were generated based on these five fields. All the models were evaluated, model 16 includes all five field parameters is the best overall performance in every analysis aspect (Table 2). Therefore, model 16 was chosen for further in-depth study.

**TABLE 2:** Statistical parameters of COMSIA models.

Field	q2	ONC	r2	F value	SEE
S, E, H, D and A	0.553	7	0.959	100.859	0.227

Model 16 was constructed using 7 components (ONC = 7) and we found cross-validated q^2^ value is 0.553, non-cross-validated r^2^ value is 0.959, standard error of estimate (SEE) is 0.227, and F-statistic is 100.859, respectively. All the values show stronger correlation between the experimental and predicted pIC50 values. The contributions of each field to the model were quantified as follows: steric field (15.6%), electrostatic field (10.1%), hydrophobic field (26.2%), hydrogen bond donor (36.0%), and hydrogen bond acceptor (12.1%). These field contributions are detailed in Table 3.

**TABLE 3:** Statistics results of COMSIA model by PLS analysis.

Model	q2	ONC	r2	SEE	F	Field contribution %				
						S	E	H	D	A
COMSIA	0.553	7	0.959	0.227	100.859	15.6	10.1	26.2	36.0	12.1

### 3.2 Validation of our COMSIA model

#### 3.2.1 Internal validation

To measure the bias of the original calculations, a bootstrapping analysis was performed over 100 runs. The results of the 100 bootstrapping analyses include the standard deviation and average R^2^ values. If the SEEboot value is smaller than SEE and the Rboot^2^ value is greater than R^2^, it proves that the constructed model is robust.

Y-randomization is a common external validation method aimed at testing whether the model’s high fitting performance is solely due to the randomness in the data by shuffling the order of the dependent variable (Y) (Király et al., 2022). This helps assess the reliability of the model (Rücker et al., 2007).

$$R_{P}^{2}=R^{2}-R_{r}^{2}$$

$R^{2}$ is the $R^{2}$ value of the original model. $R_{r}^{2}$ is the average $R^{2}$ value of the randomized models. The calculation shows that the original model’s $R^{2}$ is 0.9592, indicating a good fit on the training set. The average $R^{2}$ of the randomized models is −0.9979, meaning that after 20 rounds of Y-randomization, the models fit the training set very poorly (close to negative values). $R^{2}$ is 1.9571, representing the difference between the original model’s $R^{2}$ and the average $R^{2}$ of the randomized models. Since the $R^{2}$ value is large, it suggests that the original model has significant predictive power and that the high fitting performance is not caused by the randomness in the data. Therefore, the COMSIA model we constructed is valid and can provide reliable predictive ability. The randomization test indicates that randomness did not cause the high fitting performance of the original model, proving the model’s authenticity and validity.

#### 3.2.2 External validation

This formula is the calculation formula for the external validation coefficient $R_{ext}^{2}$ , used to assess the model’s predictive ability on an external test set (Martinez-Mayorga et al., 2024). The formula is as follows

$$R_{ext}^{2}=1-\frac{\sum_{i=1}^{ntest}{\left(y_{i}-\tilde{y_{i}}\right)}^{2}}{\sum_{i=1}^{ntest}{\left(y_{i}-{\bar{y}}_{tr}\right)}^{2}}$$

Where: ntest is the number of compounds in the external test set, $y_{i}$ is the actual bioactivity or experimental value of the i compound in the test set, $\tilde{y_{i}}$ is the predicted value of the i compound in the test set, and ${\bar{y}}_{tr}$ is the mean of the actual values in the training set (Golbraikh et al., 2003).

$R_{ext}^{2}$ is used to measure the model’s explanatory power on the external test set. How much variance the model can explain when predicting the test set. A value close to 1 indicates good predictive ability on the test set, while a value close to 0 indicates poor predictive ability. This formula compares the sum of squared errors between the predicted and actual values on the test set with the variance of the test set and normalizes it with the variance of the training set. This coefficient helps determine whether the model is overfitting the training set and ensures its generalization ability on unseen data.

Through calculation, the $R_{ext}^{2}$ value of our COMSIA model is 0.766, meaning the model has good predictive ability on the test set, explaining about 76.6% of the variability.

$R_{m\left(overall\right)}^{2}$ is the overall $R^{2}$ value, used to measure the model’s performance and goodness of fit. $R^{2}$ is the $R^{2}$ value of the training set, indicating the correlation between the predicted and actual values in the training data. $R_{0}^{2}$ is the $R^{2}$ value of the external test set (or cross-validation set), used to evaluate the model’s generalization ability on unseen data.

$$R_{m\left(overall\right)}^{2}=R^{2}\times \left(1-\sqrt{R^{2}-R_{0}^{2}}\right)$$

This formula adjusts the overall performance of the model by combining the $R^{2}$ of the training set with the $R_{0}^{2}$ of the external validation set, providing a metric that reflects both the goodness of fit on the training set and the model’s predictive ability on external data. A higher $R_{m\left(overall\right)}^{2}$ value indicates that the model not only fits well on the training set but also generalizes well to the external dataset, meaning it has strong predictive ability. The calculated $R_{m\left(overall\right)}^{2}$ is 0.530, $R^{2}$ is 0.9592, and $R_{0}^{2}$ is 0.5304. This result indicates that the model fits well on the training set (high $R^{2}$ ), and although its performance drops on the external test set, the overall $R_{m\left(overall\right)}^{2}$ value remains above 0.5, suggesting that this is a robust model.

The experimental and predicted pIC50 values, and the residual values are in Table 1. Figure 1 shows the correlation between the predicted and experimental pIC50 values for both the training and test sets which has strong and consistent relationship between them. The graphical data validate the predictive accuracy of the framework and its ability to uncover underlying patterns within the experimental dataset, thereby reinforcing its effectiveness in mechanistic pharmacological modeling.

### 3.3 COMSIA contour maps analysis

**FIGURE 2:**
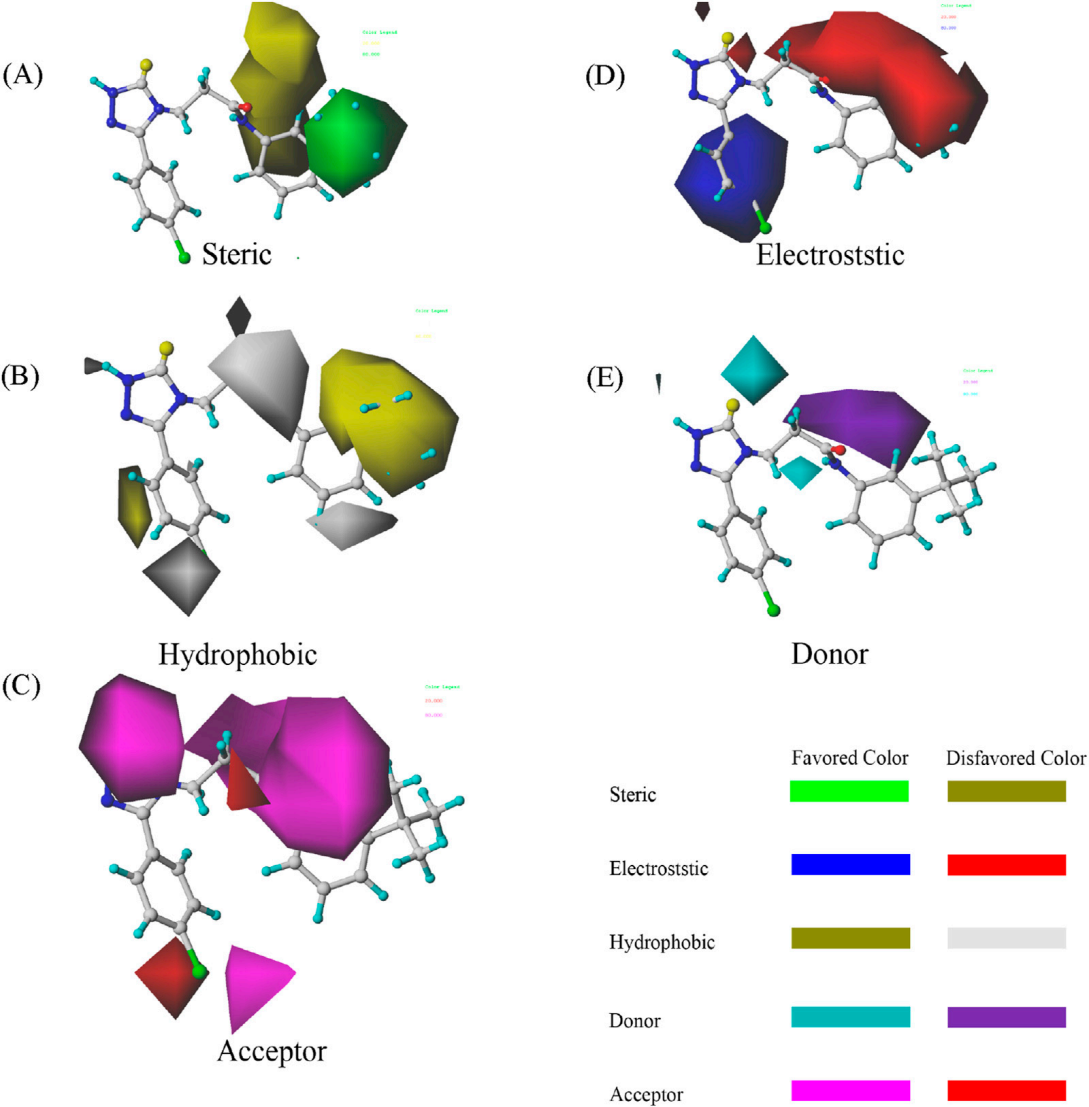
COMSIA StDev*Coeff contour maps based on the most active compound 33. **(A)** Steric **(B)** Hydrophobic **(C)** Acceptor **(D)** Electrostatic **(E)** Donor.

The COMSIA contour maps shown in Figure 2 provide valuable visual insights into the molecular fields influencing the activity of the most active compound of the 33rd. These maps represent various molecular fields, each related to specific interactions contributing to the compound’s biological activity.

The steric contours (shown in green and yellow) highlight the favorable and unfavorable spatial regions around compound 33. Positive contours (green) represent regions where large steric interactions, such as van der Waals forces, facilitate binding, while negative contours (yellow) indicate areas where steric hindrance would reduce the compound’s activity. The electrostatic field is visualized in red and blue. The positive electrostatic contours (blue) denote areas where favorable electrostatic interactions (such as hydrogen bonding or ionic interactions) with the target protein are likely to enhance binding. Conversely, negative electrostatic contours (red) represent unfavorable regions where repulsive interactions may hinder binding. Hydrophobic regions are showed in purple and cyan. Positive contours (purple) suggest regions where hydrophobic interactions are beneficial to the ligand’s binding affinity, while negative contours (cyan) represent unfavorable hydrophobic interactions that might reduce activity. The donor field is shown in pink, where positive contours (pink) indicate regions where hydrogen bond donation would be favorable for interactions with the target protein. This suggests that these regions contribute to a strong binding interaction by hydrogen bonding. The hydrogen bond acceptor field is displayed in magenta, with positive contours (magenta) signifying regions where hydrogen bond acceptance is favorable for binding to the target, thereby increasing activity. Unfavorable regions (colored red) suggest where the hydrogen bond acceptor ability may reduce activity due to poor interactions.

The contour maps provide detailed insights into how molecular modifications can enhance or impair compound activity. For compound 33, the steric field indicates opportunities for optimizing bulkiness, while the electrostatic field emphasizes the importance of carefully balancing charge distribution. The hydrophobic field strongly supports the incorporation of non-polar groups in favorable regions to enhance receptor-ligand affinity. Finally, the hydrogen bond donor and acceptor fields offer guidance on where to strategically introduce functional groups to improve hydrogen bonding interactions. By systematically applying these findings, researchers can rationally design derivatives based on compound 33 with improved activity. It can ensure that molecular modifications align with the favorable contours while avoiding the unfavorable ones. This approach underscores the power of COMSIA in guiding targeted optimization in drug design (Cramer et al., 1988).

### 3.4 Design details

We designed new structures and selected the best candidates. The first step in designing new structures involves choosing an appropriate molecular scaffold that has proven bioactivity, in this case, the scaffold shown in the image. This scaffold is typically selected based on its known interaction with the biological target, and it serves as the backbone of the new structures. The goal is to modify certain functional groups to enhance the compound’s biological activity or optimize its pharmacological properties. In the next step, a thorough analysis of the structure-activity relationship (SAR) of the current compounds (compound 33) is conducted. R1 and R2 functional groups were identified based on their impact on activity. Based on SAR, one might decide to substitute or modify certain groups (changing R1 from -Cl to -CH = CH2) to explore whether it improves the compound’s activity. Withdrawing groups (-NO_2_, -F) can enhance binding by polarizing the molecule or stabilizing charge interactions. However, electron-donating groups (-NH_2_, -OH) could improve solubility or participate in hydrogen bonding. Bulky groups (-Br, -CH = CH_2_) were evaluated for their impact on binding pocket fit. Smaller groups (-F) were prioritized in sterically constrained regions. Groups like -OH and -NH_2_ were included to exploit hydrogen bond donor/acceptor interactions with the target. Halogens (-Cl, -F, -Br) in R1 paired with polar R2 groups (-NH_2_, -OH) to enhance both binding affinity and solubility. Conjugated systems (-CH = CH_2_) paired with strong electron-withdrawing groups (-NO_2_) to optimize resonance effects. When the potential modifications were finished, all structures were incorporated into virtual screening software to predict their effects on activity. In Table 4, the compounds 33a, 33b, 33c, 33d, and 33e are the newly designed compounds with different R1 and R2 substituents. Using our built QSAR model and molecular docking, the potential biological activity (pIC50) of these compounds was predicted. The predicted pIC50 values were provided for each compound. Finally, based on the predictive models and the actual biological data, the best structures were selected for further optimization and development. In this case, the five newly designed compounds (33a, 33b, 33c, 33d, and 33e) are selected based on their predicted pIC50 values, with compound 33c having the highest predicted value of 9.674. The compound represents the optimal candidates for further research.

**TABLE 4:** The design of new structures.

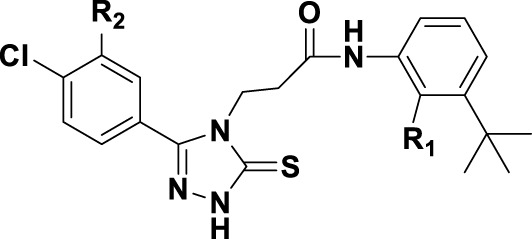			
Compd	R1	R2	Predict pIC50
33a	-CL	-NH2	9.191
33b	-CH = CH2	-NO2	9.334
33c	-F	-OH	9.674
33d	-NO2	-NH2	9.243
33e	-Br	-NO2	9.342

### 3.5 The selected new structure docking result

**FIGURE 3:**
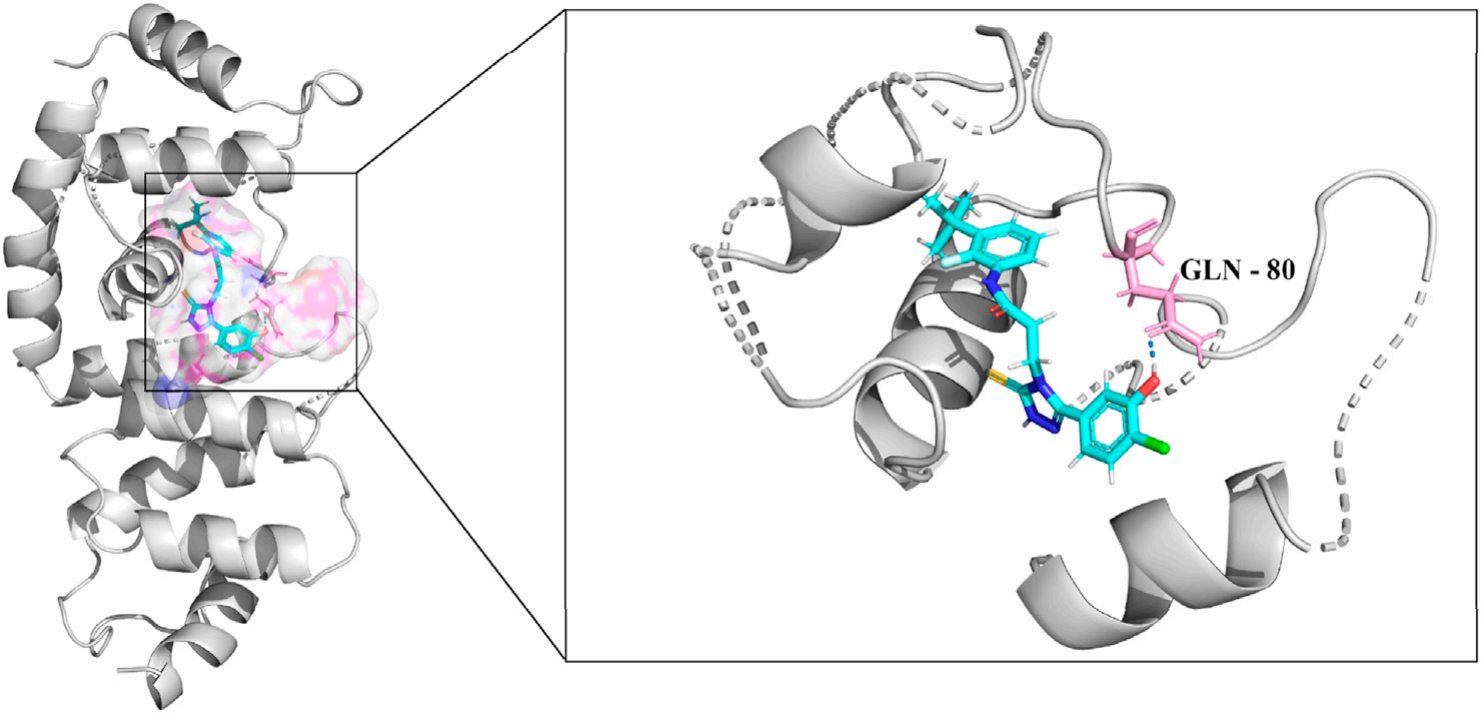
Binding interactions and molecular docking analysis of compound 33c with the target protein (PDB ID: 5UFI). The left figure shows the molecular surface of the protein with the docked compound, while the right figure provides a detailed view of the interactions between compound 33c and key residues within the binding pocket.

The molecular docking results for compound 33c (Figure 3) reveal critical insights into its binding mode and interactions with the target protein. This score represents the overall binding affinity of the compound to the protein. A positive value typically indicates favorable binding, and the magnitude suggests moderate to strong interaction. This penalizes steric clashes between the ligand and protein. The slight negative value implies minor unfavorable collisions, but they are not significant enough to influence binding. The positive value highlights the contribution of polar contacts, such as the hydrogen bond with GLN-80, which can stabilize the ligand in the binding pocket. Therefore, Figure 3 indicates the structural similarity of the docked pose to a reference conformation. A low value of IC50 suggests flexibility in the ligand’s binding mode, potentially allowing adaptive interactions with the protein. The right panel of Figure 3 details the interactions between compound 33c and residues in the binding pocket. The interaction with GLN-80 is critical for anchoring the ligand. The glutamine residue likely donates or accepts a hydrogen bond from a functional group (-OH, -NH_2_) on 33c, enhancing binding specificity and stability. The molecular surface (left panel) shows that 33c fits into the binding pocket. Non-polar regions of the ligand may engage in hydrophobic interactions with residues, while polar/charged groups align with hydrophilic residues. Depending on the ligand’s structure, aromatic rings or halogen atoms (-Cl, -F) might interact with aromatic residues.

### 3.6 Molecular dynamics simulation

The root-mean-square deviation (RMSD) trajectory reveals distinct phases of conformational behavior (Maiorov and Crippen, 1994). During the initial equilibration phase (0–2,000 frames), the RMSD rises sharply to ∼2.0 Å, reflecting structural adjustments as the system settles into a stable configuration. Between 2,000 and 10,000 frames, the RMSD oscillates within a moderate range (1.5–2.5 Å), indicating dynamic exploration of conformational states while maintaining overall structural integrity. A subtle upward trend in the red regression line suggests a gradual shift in the complex’s conformation over time. Toward the simulation’s conclusion, the RMSD peaks at ∼3.0 Å, though fluctuations remain bounded and non-catastrophic. Critically, the absence of large deviations (>3.0 Å) confirms that the complex retains its core architecture throughout the simulation, with no evidence of major destabilization or unfolding events.

**FIGURE 4:**
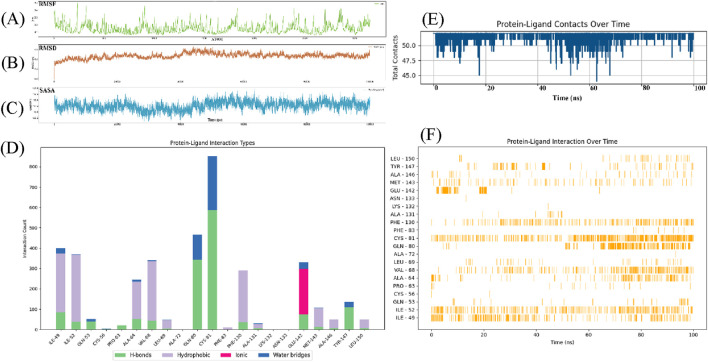
Root mean square deviation (rmsd) analysis of compound 33c in complex with target protein (PDB ID: 5UFI) during molecular dynamics simulation. **(A)** Root mean square fluctuation over time. **(B)** Root mean square deviation over time. **(C)** Solvent accessible surface area over time. **(D)** Protein-ligand interaction types (Bar plot showing different interaction types such as H-bonds, hydrophobic, ionic, and water bridges). **(E)** Protein-ligand contacts over time. **(F)** Protein-ligand interaction over time.

The RMSF profile exhibits significant variations along the atom index, displaying distinct peaks and troughs. Specific regions demonstrate elevated RMSF values (>0.2 nm), indicative of enhanced structural flexibility, while other segments show relatively lower fluctuations (0.1–0.2 nm), suggesting greater structural rigidity (Figure 4A). There is no strictly monotonic trend, suggesting that the fluctuation is uneven across the different atoms. RMSF is a key indicator of the flexibility of atoms in a molecular dynamics simulation. Higher RMSF values typically suggest more movement or flexibility in the system. The protein regions might correspond to loops, flexible linkers, or areas involved in conformational changes, which could indicate regions with more structural flexibility or less interaction with molecule. Conversely, lower RMSF values indicate regions with more rigidity or structural constraints which might correspond to the protein core or structured domains. RMSD analysis with protein-ligand interaction metrics to evaluate the stability and binding behavior of compound 33c during a molecular dynamics (MD) simulation. The RMSD rises sharply from 0 to ∼0.3nm, reflecting structural adjustments as the ligand-protein complex transitions from its docked pose to a dynamically equilibrated state. This phase involves reorientation of the ligand within the binding pocket and minor conformation changes in the protein. RMSD fluctuates between 0.15 and 0.25 nm indicate that the system maintains structural integrity. These fluctuations suggest a balance between flexibility and stability. A gradual upward drift in RMSD is observed, likely due to minor shifts in ligand positioning or protein side-chain rearrangements. The hydrogen bond with GLN-80 persists throughout the simulation, acting as an anchor to stabilize the ligand. This interaction correlates with periods of lower RMSD. It roles in maintaining structural coherence (Figure 4B). The Solvent Accessible Surface Area (SASA) data reflects the solvent indicate the protein during the simulation. It is used to assess the structural changes of the protein after ligand binding. SASA gradually decreases from approximately 117.5 nm^2^ at the start of the simulation, which shows the protein begins to contract and the reducing solvent exposure. Within the first 50 ps, SASA decreases to about 112.5 nm^2^, it is the protein initial contraction or conformational adjustment. The SASA value stabilizes after 150 ps, approximately 110 nm^2^, which indicate that the protein-ligand complex has reached a stable state. The SASA data suggests that ligand binding induces a contraction in the protein structure. This might be related to conformational changes after ligand binding. The decrease in SASA typically indicates that the binding pocket is effectively closed off and reduce solvent exposure. This suggests that the ligand stabilizes the protein structure through hydrophobic interactions or hydrogen bonding. Consistent with previous studies, this reduction in solvent exposure is usually a marker of the stability of the protein-ligand complex (Figure 4C).

The protein-ligand interaction types illustrates the distribution of different interaction types between the protein and its ligand (Figure 4D). The x-axis lists the individual amino acid residues, and the y-axis shows the count of interactions. GLN-80 and CYS-81 have notable hydrogen bond counts, reaching over 300 interactions. It has significant contributors to ligand binding (green). ILE-49 and ILE-52 have high hydrophobic interaction counts (purple), so it has contribute to the stabilization of the protein-ligand complex via hydrophobic forces. GLU-142 stands out with high number of ionic interactions, which can be seen by the large pink bar extending far beyond other residues (pink). CYS-81 also participates in water bridge interactions, but the count is generally lower than the hydrogen bond and hydrophobic interactions. They might play a secondary role in stabilizing the protein-ligand complex (blue). This chart provides valuable insights into the nature of interactions between the ligand and the protein. The data highlights that hydrophobic interactions play a central role in the protein-ligand binding, with residues such as CYS-81 contributing significantly to the stability of the complex. These residues are often involved in non-polar interactions that help to anchor the ligand within the binding pocket. Hydrogen bonds also contribute significantly to the stability of the protein-ligand complex, particularly CYS-81, which are involved in maintaining the ligand in place through polar interactions. The hydrogen bond interactions likely provide specificity to the binding. The GLU-142 indicates that electrostatic interactions are important for stabilizing the binding, particularly at the surface of the binding site where charged groups can interact with opposite charges on the ligand. The very high interaction count for GLU-142 suggests a strong electrostatic attraction that might be crucial for the stability and affinity of the ligand. Lastly, water bridges, while less prominent in this analysis, can also contribute to the fine-tuning of the protein-ligand interactions by mediating indirect interactions. They may be important for the overall solvation and flexibility of the binding site. The combination of these interaction types suggests that the ligand binds through a diverse array of forces, with a balance between hydrophobic interactions, hydrogen bonding, ionic interactions, and water bridges, which together enhance the binding stability and specificity. It is important for understanding the mechanisms of ligand binding and can guide the optimization of 1,2,4-Triazole-3-thione derivatives for improved efficacy as DCN1 inhibitors in therapeutic applications, including anticardiac fibrosis.

The total contacts between the protein and ligand over a 100 ns period, with the x-axis representing time (ns) and the y-axis representing the total contacts (Figure 4E). The protein-ligand interaction is mostly stable throughout the simulation. There are sharp decreases in contact counts, particularly at 20 ns, 50 ns, and 80 ns, where the number of contacts briefly dips below 45 contacts before recovering. These dips suggest that the ligand may undergo temporary loss or disruption of interactions with the protein during these time intervals. Despite the fluctuations, the number of contacts remains mostly consistent, with minor deviations. This indicates that the ligand maintains a relatively stable interaction with the protein, despite the transient drops in contact count. The periods of stability may represent times when the ligand is securely bound to the protein, engaging in interactions such as hydrogen bonding, hydrophobic interactions, or ionic contacts. The sharp decreases in contact counts at several points (20 ns, 50 ns, and 80 ns) could indicate transient dissociation of the ligand-protein complex. These may indicate moments when the ligand momentarily rearranges its binding conformation. The overall trend of maintaining around 50 contacts supports the hypothesis that the ligand binds stably to the protein for most of the simulation. While occasional disruptions occur, the ligand remains largely associated with the protein. This is an indicator of potential high binding affinity. This dynamic stable interaction is often observed in drug design, where small molecules engage in temporary reorganization as they stabilize their interactions over time.

The protein-ligand interactions over time (Figure 4F), where the x-axis corresponds to time (ns) and the y-axis lists the specific residues involved in the interactions. Each vertical line represents an interaction between the ligand and a particular residue at a given time during the simulation. ILE-49 and ILE-52 show consistently frequent interactions with the ligand at multiple time points, particularly in the 0–50 ns range. CYS-81 also shows significant and frequent interactions, with continuous presence across the simulation, peaking at multiple intervals. This graph clearly illustrates the dynamic nature of the protein-ligand interaction over the 100 ns molecular dynamics simulation. The frequent interactions observed for residues of ILE-49 and CYS-81 suggest that these residues are likely involved in hydrophobic interactions or covalent bonding with the ligand. ILE-49 and CYS-81 play a critical role in stabilizing the protein-ligand complex. These interactions indicate a dynamic binding mechanism, where the ligand frequently repositions itself and interacts with different residues. It is a flexibility in the binding process. This flexibility is beneficial in drug design. It allows for better accommodation of the ligand within the binding site. It can enhance the stability and potential efficacy of the inhibitor.

**FIGURE 5:**
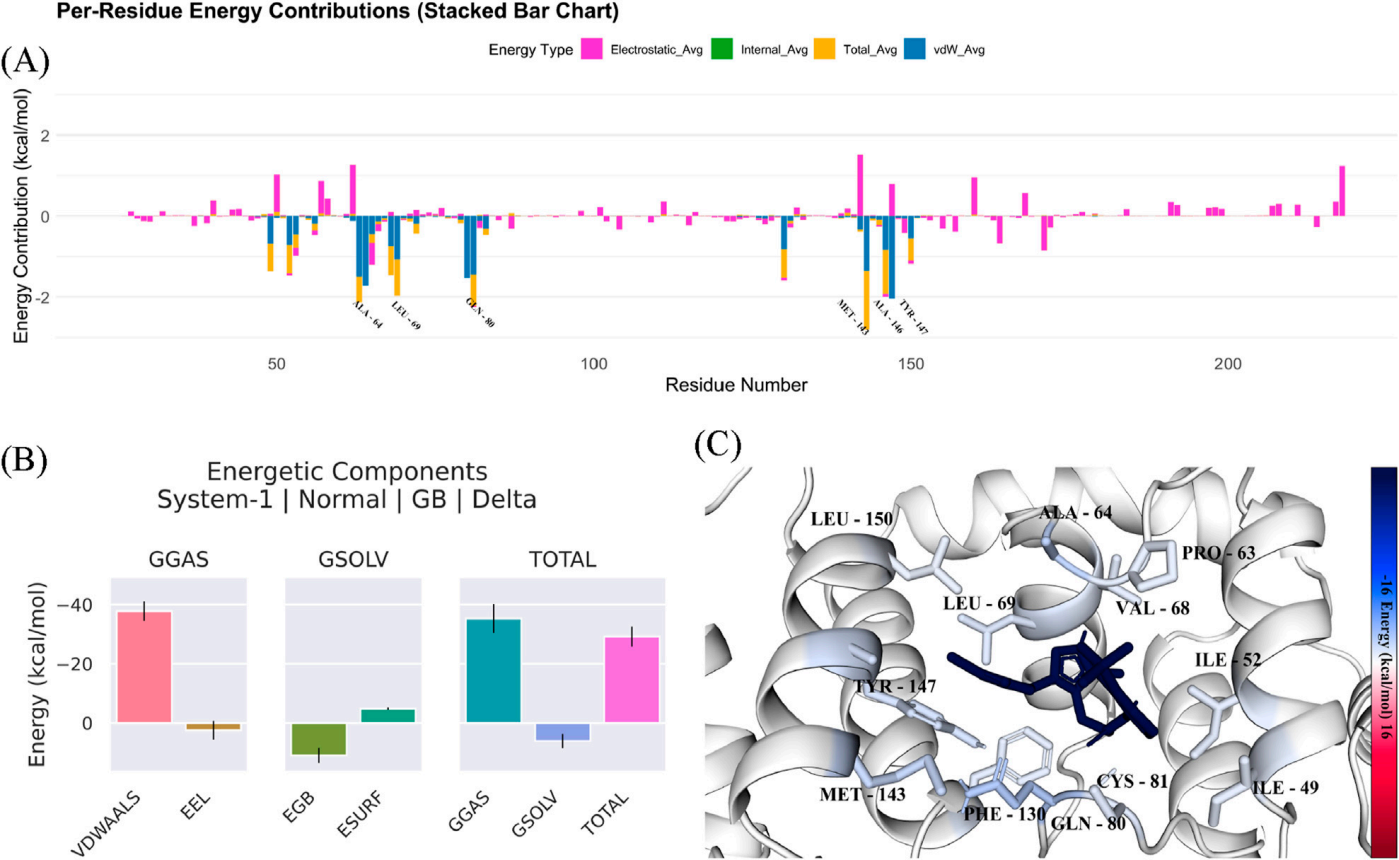
**(A–C)** Energetic components analysis of compound 33c in complex with target protein (PDB ID: 5UFI) from molecular dynamics simulation.

Figure 5 shows a stacked bar chart that provides a breakdown of energy contributions from various components across different residues of the protein-ligand complex. The energy components are categorized as electrostatic energy (pink), hydrogen bonds (yellow), Van der Waals energy (purple) and solvation energy (blue). The energy contributions for each residue helps to identify how much each residue contributes to the overall stability of the complex. Residues with high energy contributions in one or more of these categories are play key roles in stabilizing the ligand within the binding site. We found several residues with high electrostatic energy contributions (pink). These residues are involved in strong electrostatic interactions with the ligand. There were also several peaks in the Van der Waals energy (purple), which indicates that hydrophobic interactions are significant for the stability of the complex. The total energy difference (represented in pink) is about −29.18 kcal/mol, which show a favorable interaction between the ligand and protein. The standard deviation (SD) of 3.93 kcal/mol and the standard error of the mean (SEM) of 0.07 kcal/mol indicate the variability and precision of the energy values. The negative ΔTOTAL indicates that the ligand binding stabilizes the system. The protein-ligand interaction is energetically favorable. Table 5 provides the energy decomposition analysis for compound 33c bound to the target protein. It shows the average energy for each component, along with the associated standard deviation and standard error of the mean. The per-residue energy contributions (Figure 5) indicates that several residues play pivotal roles in the protein-ligand interaction, especially those contributing high values in the electrostatic and Van der Waals energy categories. These interactions suggest that electrostatic forces and hydrophobic interactions are critical for stabilizing the binding of the ligand to the protein. The identification of residues contributing significantly to these energy components can guide further optimization in drug design by focusing on enhancing these interactions to improve binding affinity. These findings demonstrate that compound 33c could be a promising candidate for targeting the protein, as it stabilizes the complex and is likely to have high binding affinity, which is crucial for therapeutic efficacy in the context of DCN1 inhibition and anticardiac fibrosis.

**TABLE 5:** Energy decomposition analysis for the binding of compound 33c with the target protein.

Delta (complex - receptor - ligand)					
Energy component	Average	SD (Prop.)	SD	SEM (Prop.)	SEM
△TOTAL	−29.18	3.93	3.44	0.07	0.06

## 4 Conclusion

In this study, we have successfully employed a combination of 3D-QSAR modeling, molecular docking, and molecular dynamics simulations to design and screen novel 1,2,4-Triazole-3-thione derivatives as potential inhibitors of DCN1 for the treatment of anticardiac fibrosis. Our COMSIA model, developed using a dataset of 47 derivatives, demonstrated robust predictive capability, with a cross-validated q2 of 0.553 and a non-cross-validated r2 of 0.959, validating its accuracy in predicting the biological activity of the compounds. The top compound 33c from our virtual screening showed a predicted pIC50 of 9.674 and displayed strong binding affinity in molecular docking simulations, as well as excellent stability in molecular dynamics simulations. Energy decomposition analysis revealed critical residues that play a pivotal role in stabilizing the ligand-protein complex, providing valuable insights for future ligand optimization and enhancing the design of more potent inhibitors. This study found that the potential of DCN1 can be an attractive therapeutic target for anticardiac fibrosis and demonstrates the effectiveness of using integrated computational techniques in the rational design of drug candidates. This findings also provide a strong foundation for the development of novel DCN1 inhibitors, which could pave the way for further research and clinical application in the treatment of cardiac fibrosis.

## Data Availability

The original contributions presented in the study are included in the article/supplementary material, further inquiries can be directed to the corresponding author.

## References

[B1] Akamatsu M. (2002). Current state and perspectives of 3D-QSAR. Curr. Top. Med. Chem. 2 (12), 1381–1394. 10.2174/1568026023392887 12470286

[B2] Buch I. Harvey M. J. Giorgino T. Anderson D. P. De Fabritiis G. (2010). High-throughput all-atom molecular dynamics simulations using distributed computing. J. Chem. Inf. Model. 50 (3), 397–403. 10.1021/ci900455r 20199097

[B3] Chatzifrangkeskou M. Le Dour C. Wu W. Morrow J. P. Joseph L. C. Beuvin M. (2016). ERK1/2 directly acts on CTGF/CCN2 expression to mediate myocardial fibrosis in cardiomyopathy caused by mutations in the lamin A/C gene. Hum. Mol. Genet. 25 (11), 2220–2233. 10.1093/hmg/ddw090 27131347 PMC5081054

[B4] Cramer R. D. Bunce J. D. Patterson D. E. Frank I. E. (1988). Crossvalidation, bootstrapping, and partial least squares compared with multiple regression in conventional QSAR studies. Quant. Structure‐Activity Relat. 7 (1), 18–25. 10.1002/qsar.19880070105

[B5] Fan J. Fu A. Zhang L. (2019). Progress in molecular docking. Quant. Biol. 7, 83–89. 10.1007/s40484-019-0172-y

[B6] Fang Y. Yu B. Liao G. Liu H.-M. (2019). “Targeting the DCN1–UBC12 protein–protein interaction: novel approaches and future directions. Future Med. Chem. 11, 2869–2871. 10.4155/fmc-2019-0253 31668094

[B7] Frangogiannis N. G. (2017). The extracellular matrix in myocardial injury, repair, and remodeling. J. Clin. investigation 127 (5), 1600–1612. 10.1172/JCI87491 PMC5409799 28459429

[B8] Frangogiannis N. G. (2021). Cardiac fibrosis. Cardiovasc. Res. 117 (6), 1450–1488. 10.1093/cvr/cvaa324 33135058 PMC8152700

[B9] Golbraikh A. Shen M. Xiao Z. Xiao Y.-D. Lee K.-H. Tropsha A. (2003). Rational selection of training and test sets for the development of validated QSAR models. J. computer-aided Mol. Des. 17, 241–253. 10.1023/a:1025386326946 13677490

[B10] He Z. Yuan Z. Yang F. Zhang J. Zhao W. Qin T. (2024). A comprehensive review on DCN1 protein, inhibitors and their therapeutic applications. Int. J. Biol. Macromol. 277, 134541. 10.1016/j.ijbiomac.2024.134541 39111501

[B11] He Z.-X. Gao G. Qiao H. Dong G.-j. Dan Z. Li Y.-l. (2024). Discovery of 1, 2, 4-Triazole-3-thione derivatives as potent and selective DCN1 inhibitors for pathological cardiac fibrosis and remodeling. J. Med. Chem. 67 (21), 18699–18723. 10.1021/acs.jmedchem.4c00713 39158077

[B12] He Z.-X. Yang W.-g. Zengyangzong D. Gao G. Zhang Q. Liu H.-M. (2023). Targeting cullin neddylation for cancer and fibrotic diseases. Theranostics 13 (14), 5017–5056. 10.7150/thno.78876 37771770 PMC10526667

[B13] Huang J. Rauscher S. Nawrocki G. Ran T. Feig M. De Groot B. L. (2017). CHARMM36m: an improved force field for folded and intrinsically disordered proteins. Nat. methods 14 (1), 71–73. 10.1038/nmeth.4067 27819658 PMC5199616

[B14] Huang X. Liu X. Li X. Zhang Y. Gao J. Yang Y. (2023). Cullin-associated and neddylation-dissociated protein 1 (CAND1) alleviates NAFLD by reducing ubiquitinated degradation of ACAA2. Nat. Commun. 14 (1), 4620. 10.1038/s41467-023-40327-5 37528093 PMC10394019

[B15] Király P. Kiss R. Kovács D. Ballaj A. Tóth G. (2022). The relevance of goodness‐of‐fit, robustness and prediction validation categories of OECD‐QSAR principles with respect to sample size and model type. Mol. Inf. 41 (11), 2200072. 10.1002/minf.202200072 PMC9787734 35773201

[B16] Klebe G. Abraham U. (1999). Comparative molecular similarity index analysis (CoMSIA) to study hydrogen-bonding properties and to score combinatorial libraries. J. computer-aided Mol. Des. 13, 1–10. 10.1023/a:1008047919606 10087495

[B17] Lanka G. Begum D. Banerjee S. Adhikari N. Ghosh B. (2023). Pharmacophore-based virtual screening, 3D QSAR, Docking, ADMET, and MD simulation studies: an *in silico* perspective for the identification of new potential HDAC3 inhibitors. Comput. Biol. Med. 166, 107481. 10.1016/j.compbiomed.2023.107481 37741229

[B18] Li W. Si H. Li Y. Ge C. Song F. Ma X. (2016). 3D-QSAR and molecular docking studies on designing inhibitors of the hepatitis C virus NS5B polymerase. J. Mol. Struct. 1117, 227–239. 10.1016/j.molstruc.2016.03.073

[B19] Lu Y. Yang X. (2020). The pivotal roles of neddylation pathway in immunoregulation. Immun. Inflamm. Dis. 8 (4), 782–792. 10.1002/iid3.335 32749072 PMC7654410

[B20] Maiorov V. N. Crippen G. M. (1994). Significance of root-mean-square deviation in comparing three-dimensional structures of globular proteins. J. Mol. Biol. 235 (2), 625–634. 10.1006/jmbi.1994.1017 8289285

[B21] Martinez-Mayorga K. Rosas-Jiménez J. G. Gonzalez-Ponce K. López-López E. Neme A. Medina-Franco J. L. (2024). The pursuit of accurate predictive models of the bioactivity of small molecules. Chem. Sci. 15 (6), 1938–1952. 10.1039/d3sc05534e 38332817 PMC10848664

[B22] Morris G. M. Lim-Wilby M. (2008). Molecular docking. Mol. Model. proteins 443, 365–382. 10.1007/978-1-59745-177-2_19 18446297

[B23] Paccez J. D. Foret C. L. de Vasconcellos J. F. Donaldson L. Zerbini L. F. (2024). DCUN1D1 and neddylation: potential targets for cancer therapy. Biochimica Biophysica Acta (BBA)-Molecular Basis Dis. 1870, 167308. 10.1016/j.bbadis.2024.167308 38885797

[B24] Putz M. V. Duda-Seiman C. Duda-Seiman D. Putz A.-M. Alexandrescu I. Mernea M. (2016). Chemical structure-biological activity models for pharmacophores’ 3D-interactions. Int. J. Mol. Sci. 17 (7), 1087. 10.3390/ijms17071087 27399692 PMC4964463

[B25] Rücker C. Rücker G. Meringer M. (2007). y-Randomization and its Variants in QSPR/QSAR. J. Chem. Inf. Model 47, 2345–2357. 10.1021/ci700157b 17880194

[B26] Segura A. M. Frazier O. Buja L. M. (2014). Fibrosis and heart failure. Heart Fail. Rev. 19, 173–185. 10.1007/s10741-012-9365-4 23124941

[B27] Valdés-Tresanco M. S. Valdés-Tresanco M. E. Valiente P. A. Moreno E. (2021). gmx_MMPBSA: a new tool to perform end-state free energy calculations with GROMACS. J. Chem. theory Comput. 17 (10), 6281–6291. 10.1021/acs.jctc.1c00645 34586825

[B28] Vanommeslaeghe K. MacKerell A. D. Jr (2012). Automation of the CHARMM General Force Field (CGenFF) I: bond perception and atom typing. J. Chem. Inf. Model. 52 (12), 3144–3154. 10.1021/ci300363c 23146088 PMC3528824

[B29] Zheng Y.-C. Guo Y.-J. Wang B. Wang C. Mamun M. A. A. Gao Y. (2021). Targeting neddylation E2s: a novel therapeutic strategy in cancer. J. Hematol. and Oncol. 14 (1), 57. 10.1186/s13045-021-01070-w 33827629 PMC8028724

